# Exosomes From M2 Macrophage Promote Peritendinous Fibrosis Posterior Tendon Injury via the MiR-15b-5p/FGF-1/7/9 Pathway by Delivery of circRNA-Ep400

**DOI:** 10.3389/fcell.2021.595911

**Published:** 2021-08-27

**Authors:** Yinxian Yu, Binbin Sun, Zhuoying Wang, Mengkai Yang, Zhi Cui, Subin Lin, Mingming Jin, Chengqing Yi

**Affiliations:** ^1^Department of Orthopaedic Surgery, Shanghai General Hospital, Shanghai Jiao Tong University School of Medicine, Shanghai, China; ^2^Department of Orthopaedic Surgery, Shanghai Ninth People’s Hospital, Shanghai Jiao Tong University School of Medicine, Shanghai, China; ^3^Department of Orthopedics, The Second Affiliated Hospital of Soochow University, Suzhou, China; ^4^Shanghai Key Laboratory of Molecular Imaging, Shanghai University of Medicine and Health Sciences, Shanghai, China

**Keywords:** M2 macrophage, exosomes, circRNA-Ep400, tendon injury, miR-15b-5p, FGF-1/7/9

## Abstract

Achilles tendon rupture prognosis is usually unsatisfactory. After the tendon is injured, it may not function properly because of the fibrotic healing response, which restrains tendon motion. Inflammatory monocytes and tissue-resident macrophages are indispensable regulators in tissue repair, fibrosis, and regeneration. Exosomes from macrophages are crucial factors in tissue microenvironment regulation following tissue injury. This study therefore aimed to clarify the roles of macrophage exosomes in tendon injury (TI) repair. The results show that macrophages play a role after TI. M1 macrophages were increased relative to peritendinous fibrosis after TI. High-throughput sequencing showed abnormal expression of circular RNAs (circRNAs) between exosomes from M2 and M0 macrophages. Among the abnormal expressions of circRNA, circRNA-Ep400 was significantly increased in M2 macrophage exosomes. The results also show that M2 macrophage-derived circRNA-Ep400-containing exosomes are important for promoting peritendinous fibrosis after TI. Bioinformatics and dual-luciferase reporting experiments confirmed that miR-15b-5p and fibroblast growth factor (FGF)-1/7/9 were downstream targets of circRNA-Ep400. High circRNA-Ep400-containing exosome treatment inhibited miR-15b-5p, but promoted FGF1/7/9 expression in both fibroblasts and tenocytes. Furthermore, high circRNA-Ep400-containing exosome treatment promoted fibrosis, proliferation, and migration in both fibroblasts and tenocytes. Taken together, the results show that M2 macrophage-derived circRNA-Ep400-containing exosomes promote peritendinous fibrosis after TI via the miR-15b-5p/FGF-1/7/9 pathway, which suggests novel therapeutics for tendon injury treatment.

## Introduction

Tendon tears and tendinopathies are common musculoskeletal injuries, which contribute to > 30% of musculoskeletal consultations (McCormick et al., 1995). Flexor tendon injury (TI) usually occurs during lacerations, and the incidence is highest among patients 20–29 years of age, with a higher incidence in males than in females (de Jong et al., 2014). Of these injuries, tendinopathy has a huge socioeconomic burden, predicted to result in an annual medical cost and indirect lost salary expenditure of approximately $850 billion (Wang et al., 2018). After injury, tendons usually heal through peritendinous fibrosis, without leading to regeneration of natural tendon structure, which involves adhesion between the nearby synovial sheath and tendon, resulting in impaired tendon gliding and ultimately in tissue stiffness (Wynn and Ramalingam, 2012; Zhou et al., 2018).

Tendon disorders are common and lead to significant disability, pain, healthcare costs, and lost productivity. A wide range of injury mechanisms exist leading to tendinopathy or tendon rupture (Pitt et al., 2016). Tendon healing after surgical repair generally progresses through a short inflammatory phase, which lasts about a week; followed by a proliferative phase, which lasts a few weeks; followed by a remodeling phase, which lasts many months (Zhang et al., 2015). During the inflammatory phase, vascular permeability increases and an influx of inflammatory cells enters the healing site. These cells produce a number of cytokines and growth factors that lead to recruitment and proliferation of macrophages and resident tendon fibroblasts. During the proliferative and remodeling phases of healing, fibroblasts proliferate and begin to produce, deposit, orient, and crosslink fibrillar collagens (Thomopoulos et al., 2015). Previous studies have found the potential of macrophages to convert into collagen-producing fibroblast-like cells, in the absence of any other accessory cells. This macrophage to fibroblast-like transition might augment fibrosis by collagen deposition (Haider et al., 2019).

Recent evidence suggests that modulation of inflammation in the early stages following tendon repair may lead to improved healing (Hays et al., 2008). Macrophages play essential roles in both promoting and resolving inflammation and in both facilitating and moderating tissue repair. In an injury setting, M1 cells predominate early, whereas M2 macrophages accumulate later (Lichtnekert et al., 2013). Increasing numbers of studies have found that macrophages affect the organizational microenvironment by secretion of exosomes and transmission of molecules with biological information (Momen-Heravi et al., 2014).

Exosomes are small endocytic origin membrane vesicles generated by the majority of cells in culture. Exosomes are the main extracellular signaling pathways, which are implicated in various traits and are involved in different physiological processes (Zhang et al., 2015; Pitt et al., 2016; Thomou et al., 2017; Ying et al., 2017). They originate from fusion interactions among cells and carriers of genetic information, which function in repairing inflammation and tissue when shuttled by exosomes (Bartel, 2004; Wang C. et al., 2017; Ying et al., 2017). An increasing number of studies have reported that macrophages function in tissue repair, regeneration, and fibrosis (Wynn and Vannella, 2016). Persistent tilt polarization toward M2 macrophages correlates with fibrosis and the epithelial-mesenchymal transition (Yao et al., 2016; Wang et al., 2019). In addition, whether exosomes from M2 macrophages have an inductive effect during peritendinous fibrosis after TI is unknown. This study therefore aimed to characterize the therapeutic mechanism of M2 macrophage exosomes after TI, and to then identify the therapeutic mechanism of how M2 macrophage exosomes promote peritendinous fibrosis posterior TI.

## Materials and Methods

### Animals and Macrophage Depletion

The Animal Research Committee in Shanghai Jiao Tong University Affiliated Shanghai First People’s Hospital approved the animal protocols. We performed all procedures following NIH Guidelines regarding Laboratory Animal Care and Use.

We used phosphate-buffered saline (PBS)-Lipo or Clo-Lipo^1^ following a published protocol (Bartel, 2004). In brief, 2 days before tendon surgery, we administered six- to 8-week-old male C57BL/6 mice 1.33 mL/kg of PBS-Lipo or Clo-Lipo by intraperitoneal injection, and injected another 1.33 mL/kg of Clo-Lipo directly into the tendon during surgery. The animals received the same weekly dosage (1.33 mL/kg) of injections during the procedure. Each group was comprised of six mice.

### Flexor Tendon Surgery

The flexor digitorum longus (FDL) tendons in the mouse right hind paw were fully transected and repaired (Cui et al., 2019). In summary, we anesthetized animals by an injection of 4 mg/kg xylazine and 60 mg/kg ketamine. After surgical site preparation, we performed surgery through the FDL in the transverse plane at the calf myotendinous junction to ensure that the repair site would not be affected by high strain. We closed the skin using 6-0 nylon running sutures (Ethicon, Edinburgh, United Kingdom). Afterward, we made a 2 cm incision on the posterior hind paw surface, and retracted soft tissue to detect the FDL. We fully transected the FDL using microscissors and then used 8-0 prolene sutures (Ethicon) in a modified Kessler pattern for repair. We then closed the skin using 6-0 nylon running sutures. The sham surgery was conducted in the control group using an identical exposure protocol and anesthesia, but the isolated FDL tendon was not damaged. We then returned the mice to their cages and allowed them to move freely and bear weight after recovery from anesthesia. After allowing FDL tendon repair for 3 weeks, the mice were sacrificed by cervical dislocation and used for subsequent analyses. Each group was comprised of six mice.

### Mechanical Testing

To test the mechanical properties of the healing tendon after macrophage treatment, day 14 tendons were mechanically tested. Achilles tendons were carefully dissected and surrounding tissue excised to keep the calcaneal insertion site intact. Tendons remained hydrated using PBS. Tendon length, width, and thickness were measured using a dissecting microscope and digital calipers during the pre-load. Width and thickness measurements were obtained at the injury site. The cross sectional area (assumed to be an ellipse) was then estimated. Tendons were tested in a custom-designed load frame, which gripped and loaded the tendons along their longitudinal axis. The calcaneus was trimmed and press-fit into a custom bone grip. The soft tissue end of the specimens was fixed to strips of Tyvek (McMaster-Carr, Elmhurst, IL, United States) with adhesive (super glue; Ace Hardware, Oak Brook, IL, United States), which were held between two plates to form the soft-tissue grip. Dimensional measurements for the tendons were recorded at preload. Mechanical testing was performed at room temperature. A low preload of 0.1 N was applied in order to obtain a uniform zero point prior to preconditioning (20 cycles at 0.5 Hz) to 0.5%. Pull-to-failure testing was performed on tendons at a rate of 3.33 mm/s. Force and displacement data from the test system were recorded at 10 Hz. Failure force was the highest load prior to failure of the tendon, and Lagrangian stress was calculated by dividing the failure force by the initial cross-sectional area of the tendon. Stiffness was calculated by determining the slope of the most linear portion of the load–displacement curve. Young’s modulus was calculated using the slope of the linear portion of the stress-strain curve.

### Histological Evaluation

After euthanizing the mice, skin and excess soft tissue were removed at the mid tibia, while skin on the foot sole remained intact and did not affect the repair site. The samples were then fixed for 1 day using 4% paraformaldehyde (PFA) in PBS. After dehydration in a series of ethanol gradients and embedding in paraffin, we cut 4 μm sagittal slices, which were stained with Masson’s trichrome, F4/80, and for inducible nitric oxide synthase (iNOS).

### Cell Culture

We purchased the NIH 3T3 fibroblast cell line from the Cell Bank of the Type Culture Collection in the Chinese Academy of Sciences (Shanghai Institute of Cell Biology, Shanghai, China) and cultured them in Dulbecco’s Modified Eagle’s Medium (DMEM; Gibco, Gaithersburg, MD, United States) containing 10% fetal bovine serum (FBS; Gibco).

We harvested tenocytes from mouse superficial flexor tendons (Thomopoulos et al., 2015). In brief, we digested tendon pieces with 0.15% collagenase NB4 (SERVA Electrophoresis, Heidelberg, Germany) for 2 h at 37°C. We then filtered the mixture through cell mesh (60 μm) (Corning, Corning, NY, United States). After centrifugation at 300 × *g* for 5 min, we resuspended the cell pellet in DMEM containing 10% FBS and 1% penicillin-streptomycin.

We separated bone marrow-derived macrophages (BMDMs; M0 macrophages) from adult mice femurs and tibias. We flushed tibias, and seeded bone marrow cells into 10 cm^2^ tissue culture plates, which were cultured in alpha minimum essential medium (alpha MEM; Gibco) with 10% FBS and 1% penicillin-streptomycin at 37°C, supplemented with 10^4^ U/mL macrophage colony-stimulating factor for 1 day. We cultured BMDMs in alpha-MEM with 10% exosome-free FBS to generate exosomes. For M2 macrophage induction, 20 ng/mL IL-13 and IL-4 were used for 1 day to treat M0 macrophages as previously described (Thomou et al., 2017).

### Exosome Purification, Labeling, and Characterization

After 3 days of macrophage culture, we removed dead cells and debris in the medium by centrifugation at 1,000 × *g* for 10 min and filtered the samples using a 0.2 μm filter. We then subjected the medium to ultracentrifugation at 100,000 × *g* for 4–6 h at 4°C. After washing with PBS (100,000 × *g* for 20 min), we resuspended the exosome-containing pellet in PBS. For electron microscopy, we fixed the exosomes with 2% PFA and mounted them on a Formvar- and carbon-coated copper mesh grid. The exosomes were placed the grids on 2% gelatin for 20 min at 37°C, rinsed with 0.15 M glycine and PBS and blocked using 1% cold water fish skin gelatin. The grids were then viewed using transmission electron microscopy.

To track the exosomes, we labeled exosomes with fluorescent dye using a PKH26 fluorescent cell linker kit (Sigma-Aldrich, St. Louis, MO, United States), following standard procedures. After labeling, the PKH26-exosomes were rinsed with PBS, concentrated using ultracentrifugation (100,000 × *g* for 20 min) at 4°C and resuspended the in PBS.

### High-Throughput and Strand-Specific RNA-Seq Library Construction

Total RNA was extracted from exosomes isolated from M0 and M2 macrophages using TRIzol reagent (Invitrogen, Carlsbad, CA, United States). We obtained approximately 3 μg total RNA from every specimen using the VAHTS Total RNA-seq (H/M/R) Library Prep Kit from Illumina (Vazyme Biotech, Nanjing, China) to eliminate ribosomal RNA, but retained other RNAs like ncRNA and mRNA. We treated the purified RNA with RNase R (40 U, for 3 h at 37°C) (Epicenter), which was followed by TRIzol purification. We constructed a RNA-seq library using the KAPA Stranded RNA-Seq Library Prep Kit (Roche, Basel, Switzerland) and deep sequencing with a HiSeq 4000 (Illumina, San Diego, CA, United States) conducted by Aksomics (Shanghai, China).

### Bioinformatic Analysis

We predicted circRNA/miRNA target genes using *Circular RNA Interactome*. We predicted interactions between miR-15b-5p and FGF using^2^.

### Fluorescence *in situ* Hybridization (FISH)

We obtained specific probes to circRNA-Ep400 (Dig-5′-ACAGGTGGACCCAGAACCAGCCAG-3′-Dig) from Geneseed Biotech (Guangzhou, China). FITC-conjugated anti-biotin antibodies were also purchased from Jackson ImmunoResearch (West Grove, PA, United States). We counterstained the nuclei with 4,6-diamidino-2-phenylindole (DAPI). Finally, we obtained images using an LSM 700 confocal microscope (Carl Zeiss, Oberkochen, Germany).

### Total RNA Isolation and RT-qPCR

Total RNA was isolated from cells and tissues utilizing TRIzol reagent (Invitrogen), using standard procedures. We spectrophotometrically examined the concentration and purity of RNA samples by detecting absorbance at 230, 260, and 280 nm using a NanoDrop ND-1000 (Thermo Fisher Scientific, Wilmington, DE, United States). Specifically, we assumed that OD260/OD280 ratios of 1.8–2.1 were acceptable, and that OD260/OD230 ratios greater than 1.8 were acceptable.

We reverse transcribed total RNA previously detected using RT-qPCR. The primers specific for circRNA-Ep400 and miR-15b-5p, as well as FGF1/7/9 were obtained from GenePharma (Shanghai, China). RT-qPCR was performed using an AB7300 thermo-recycler (Applied Biosystems, Carlsbad, CA, United States) leveraging primers and the TaqMan Universal PCR Master Mix. The *GAPDH* gene was used as a reference for mRNAs and circRNAs. U6 was used as an internal control for the miRNA expression scale. Gene expression was quantified by 2^–ΔΔ*Ct*^. Primers utilized to assay circRNA-Ep400 expression included forward, 5′-GGACTTCGGAGAGCTTC-3′ and reverse, 5′-CCGTCTCCCTGTGGTCGTC-3′. The miR-15b-5p primer was, 5′-TAGCAGCACATAATGGTTTGTG-3′. The FGF1 primers were forward, 5′-CCAACCCAGGAGATCATTTG-3′ and reverse, 5′-ACCCAGCCTGACAGACAATC-3′. The FGF7 primers were forward, 5′-TGGGCACTATATCTCTAGCTTGC-3′ and reverse, 5′-GGGTGCGACAGAACAGTCT-3′. The FGF9 primers were forward, 5′-TCACTTGAGCCCTTAAAACATAT AAATGCTTTCATGCGGTG-3′ and reverse, 5′-CACCGCAT GAAAGCATTTATATGTTTTAAGGGCTCAAGTG-3′. The *U6* primers were forward, 5′-CTCGCTTCGGCAGCA CA-3′ and reverse, 5′-ACGCTTCACGAATTTGCGTGTC-3′. The *GAPDH* primers were forward, 5′-GATGAGTTCA GGCAACATC-3′ and reverse, 5′-TGGTGAAGACGCCAG TGGA-3′.

### The Dual-Luciferase Reporter Assay

The circRNA-Ep400 binding site and the FGF-1/7/9 3′-UTR, termed circRNA-Ep400-WT, circRNA-Ep400-Mut, FGF-1/7/9-3′UTR WT, and PROK2-3′-UTR-Mut were placed in a pGL3 promoter vector (Realgene, Nanjing, China) using *Kpn*I and *Hin*dIII sites in a dual-luciferase reporter assay. 293T cells were plated into 24-well plates, and then transfected 5 ng Renilla of the luciferase vector, pRL-SV40, 50 nM miR-15b-5p mimics, 80 ng plasmid, and negative control using Lipofectamine 2000 (Invitrogen). Cells were collected and assessed 2 days after transfection using a Dual-Luciferase Assay (Promega, Madison, WI, United States). The experiments were independently repeated three times.

### The 5-Ethynyl-2′-Deoxyuridine (EdU) Assay

An EdU assay kit (RiboBio, Guangzhou, China) was used to measure DNA synthesis and cell proliferation. We seeded 10,000 NIH 3T3 cells and tenocytes, which were previously treated in a 96-well plate overnight. The following day, we added 25 μM Edu solution to 96-well plate, followed by incubation and observation the next day. A solution of 4% formalin was then used to fix the cells for 2 h at room temperature. Next, 0.5% Triton X-100 was used to permeabilize the cells for 10 min. Next, 200 μL of the Apollo reaction solution was added for 30 min to stain for EdU, after which 200 μL of DAPI was added to stain the nuclei. Finally, a light microscope (Nikon, Tokyo, Japan) was used to detect cell proliferation and DNA synthesis, which was reflected by blue and red signals, respectively.

### The Transwell and Wound Healing Assays

Fibroblast and tenocyte cells were suspended in 200 μL of serum-free medium and added to an upper Transwell chamber with 8 μm pores (Corning Costar, Corning, NY, United States) We then added 600 μL of medium, including 20% FBS, to the lower chamber as a chemoattractant. After incubation for 1 day, we immersed the cells in the filter with methanol, which were then stained with 0.1% Crystal Violet. Finally, the stained cells were counted in three fields of view (200×).

### Statistical Analyses

Differences between both groups were assessed by paired or unpaired two-tailed *t*-tests. Pearson′s correlation test was used to determine associations between both groups. One-way ANOVA and the non-parametric with Kruskal-Wallis test were used for multiple groups comparisons. We expressed the results as the mean ± SEM. We considered *p* < 0.05 as significant. Prism software (GraphPad, San Diego, CA, United States) was used for all statistical analyses.

## Results

### Macrophages Play an Important Role After TI

**FIGURE 1:**
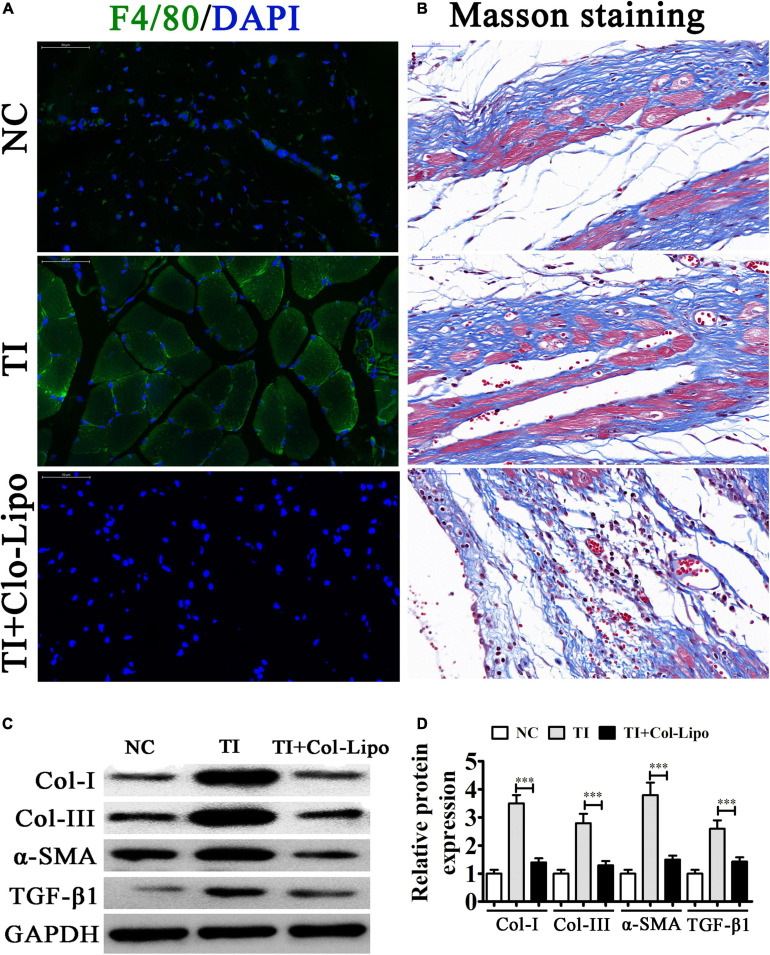
Macrophages play an important role after tendon injury (TI) with or without Clo-containing liposomes. **(A)** Immunostaining of macrophages (F4/80^+^ cells were green) in each group; the cell nuclei were stained with 4,6-diamidino-2-phenylindole (blue). **(B)** Masson’s trichrome staining showing the fibrosis of tendon tissue. **(C,D)** Western blot analysis and quantification of the Col I, Col III, and α-SMA, as well as TGF-β1 expression levels. The results are expressed as the mean ± SD, ^∗∗∗^*P* < 0.001 vs. TI.

To identify the role of BMDMs in peritendinous fibrosis after TI, we depleted macrophages using Clo-Lipo 2 days before and just prior to tendon surgery (days 2 and 0, respectively) and at 1, 2, and 3 weeks following TI, while the control group received PBS-containing liposomes. We allocated the mice into the three groups in a random manner involving the sham operation (NC), and FDL TI, and TI with clodronate liposomes (Clo-Lipo) treatment (TI-Clo). The results of the immunofluorescence detection showed that F4/80 expression was increased after TI, suggesting that macrophages had infiltrated into the damaged tissues. In contrast, Clo-Lipo pretreatment suppressed macrophage infiltration (Figure 1A). Masson staining showed that fibrogenesis was increased at 21 days after TI. Macrophage depletion (TI + Clo-Lipo group) decreased fibrogenesis, suggesting that macrophage depletion efficiently alleviated peritendinous fibrosis, revealing the role of macrophages in the development of tendon adhesion (Figure 1B). Western blot analysis showed that macrophage depletion inhibited peritendinous fibrosis by decreasing the accumulation of extracellular matrix (ECM) components such as collagen type I (COL I), COL III, and alpha smooth muscle actin (α-SMA) in injured tendons, as well as TGF-β1 (Figures 1C,D).

### Macrophage Phenotypic Transformation Plays a Role in Regulating Peritendinous Fibrosis After TI

**FIGURE 2:**
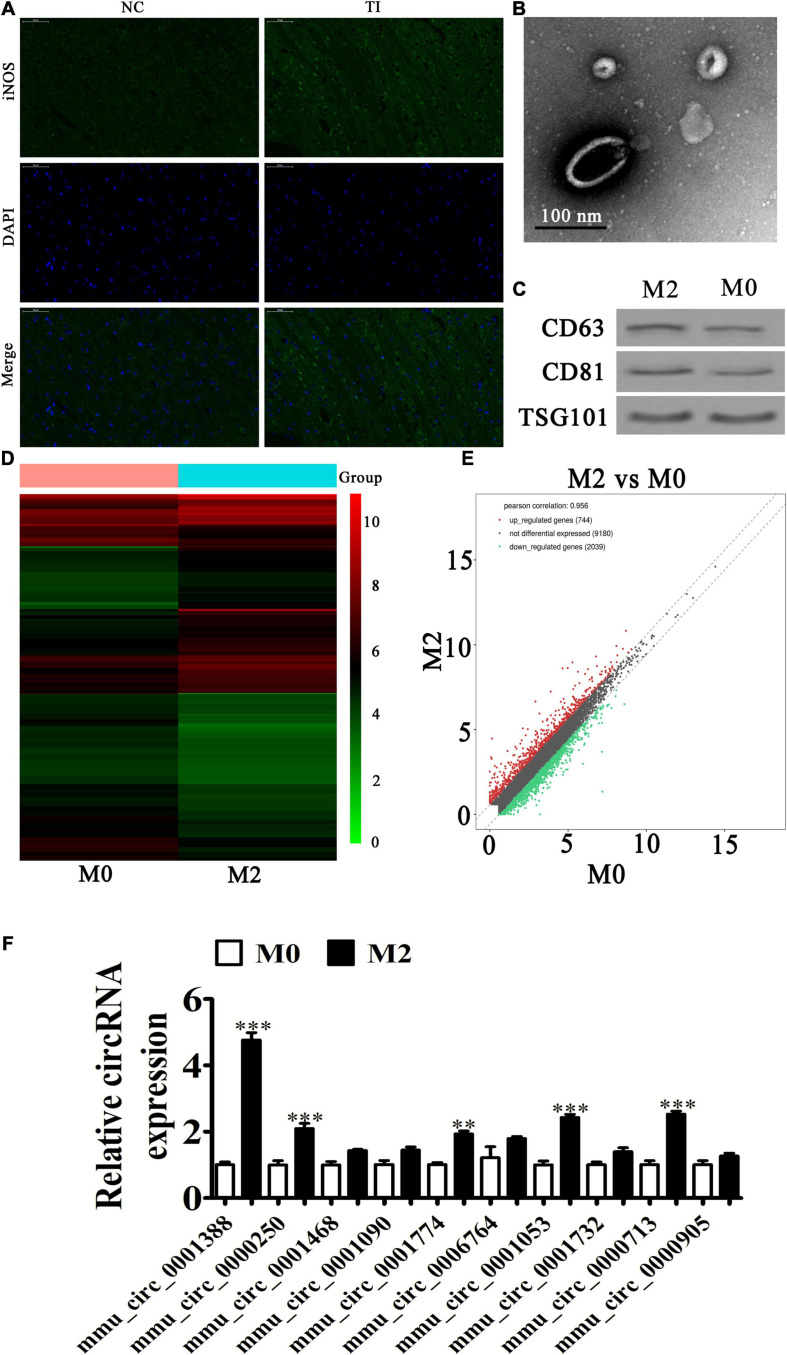
Macrophage phenotypic transformation plays a role in regulating peritendinous fibrosis after tendon injury (TI). **(A)** Immunofluorescence detection showing the expression of iNOS (M1 marker). **(B)** Transmission electron microscopy shows exosome secretion from macrophages. **(C)** Western blot analysis showing the expression of exosome specificity markers (CD61, CD81, and TSG101). **(D)** A heat map shows the differentially expressed circRNAs. **(E)** A volcano plot showing the upregulation and downregulation of circRNA from M0 and M2 macrophage exosomes. **(F)** RT-qPCR detection comparing the upregulated expression of ten circRNAs from M0 and M2 macrophage exosomes. The results are expressed as the mean ± SD, ^∗∗^*P* < 0.01, ^∗∗∗^*P* < 0.001 vs. M0 macrophages.

Our immunofluorescence study also showed that iNOS (M1 macrophage marker) expression during peritendinous was increased after TI (Figure 2A). The result also showed that M2 macrophages showed almost no difference between the NC and TI groups using CD206 staining (red) (Supplementary Figure 1). Other studies have reported that persistent tilt polarization toward M2 macrophages correlated with fibrosis and the epithelia-mesenchymal transition (Wang Y. Y. et al., 2017; Wang et al., 2019; Lu et al., 2018). To determine if M2 macrophages promoted peritendinous fibrosis posterior TI by secreting exosomes, IL-4 and IL-13 at 20 ng/mL was used for M2 macrophage induction. Electron microscopy results suggested abundant macrophage-derived exosomes having diameters of 70–150 nm (Figure 2B). Western blotting showed that the vesicles were positive for the exosome-specific markers of CD63, CD81, and TSG101 (Figure 2C), confirming that the structures were mainly exosomes, and that they were derived from macrophages.

To identify the regulatory mechanism, exosomes from M0 and M2 macrophages were used for high-throughput RNA-Seq. The result showed that there were 744 upregulated and 2,039 downregulated circRNAs in M2 macrophage exosomes, when compared with M1 macrophage exosomes (Figures 2D,E and Supplementary Figure 1). We selected ten upregulated circRNAs for RT-qPCR detection. The results showed that mmu_circ_0001388 (circRNA-Ep400) significantly increased (approximately fivefold) (Figure 2F), suggesting that circRNA-Ep400 played a role in M2 macrophage exosome-mediated promotion of peritendinous fibrosis following TI.

### M2 Macrophage-Derived circRNA-Ep400-Containing Exosomes Function in Promoting Peritendinous Fibrosis After TI

**FIGURE 3:**
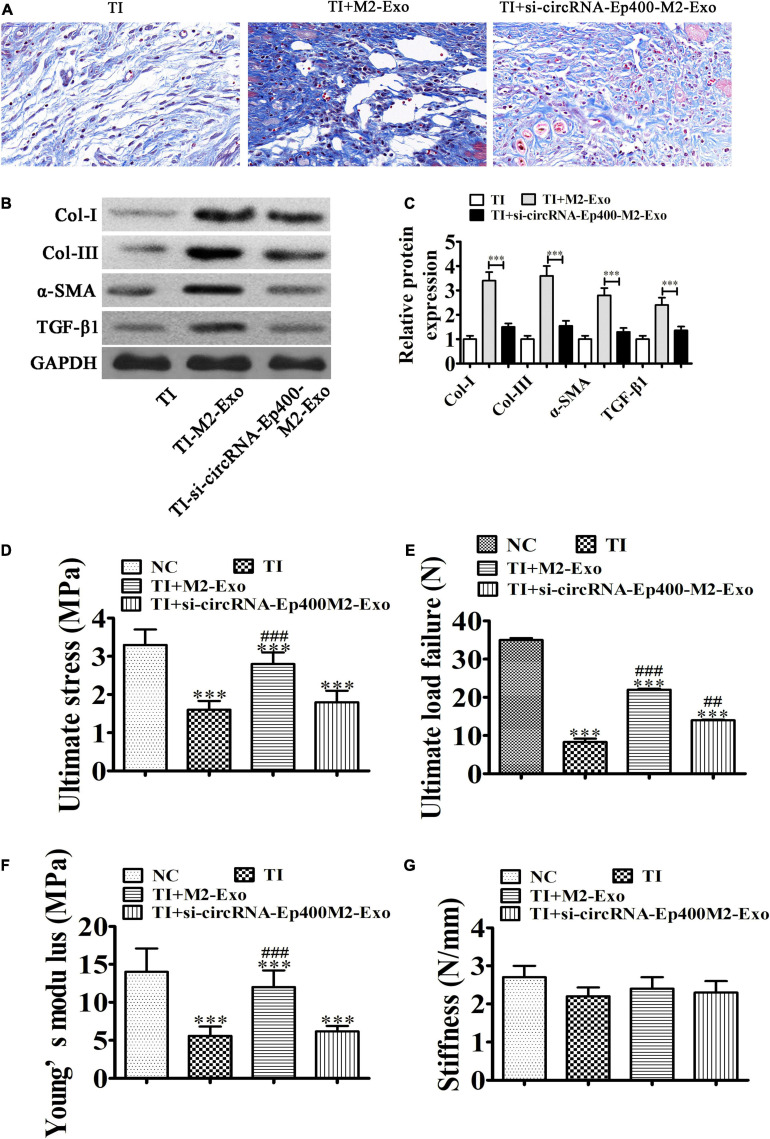
M2 macrophage-derived circRNA-Ep400-containing exosomes function in promoting peritendinous fibrosis after tendon injury. **(A)** Masson’s trichrome staining showing the fibrosis of tendon tissue. **(B,C)** Western blot analysis and quantification of the Col I, Col III, and α-SMA, as well as TGF-β1 expression levels. The results are expressed as the mean ± SD, ^∗∗∗^*P* < 0.001. **(D–G)** The tensile strengths of tendons after impairment were measured, including ultimate stress **(D)**, ultimate load failure **(E)**, Young’s modulus **(F)**, and stiffness **(G)**. The results are expressed as the mean ± SD. ^∗∗∗^*P* < 0.001 vs. NC. ^##^*P* < 0.01, ^###^*P* < 0.001 vs. TI.

Masson’s staining showed that fibrogenesis was increased 21 days after TI and M2 macrophage exosome treatment. However, exosomes from circRNA-Ep400-downregualted M2 macrophages effectively alleviated peritendinous fibrosis (Figure 3A). Western blotting showed that circRNA-Ep400 depletion of M2 macrophage exosomes inhibited peritendinous fibrosis by decreasing ECM component accumulations including Col-I, Col-III, and α-SMA in injured tendons, as well as TGF-β1 (Figures 3B,C), suggesting that M2 macrophage-derived circRNA-Ep400-containing exosomes played an important role in the induction of peritendinous fibrosis as a result of TI. Biomechanical tests can be used to evaluate the tensile strength of tendons after impairment. The mean tensile strength of normal tendons was 34.32 ± 2.24. The tensile strength had decreased during 3 weeks. M2 macrophage exosome treatment increased the tensile strength of tendons, but after downregulation of circRNA-Ep400, the therapeutical effect was inhibited. The results also showed that M2 macrophage exosome treatment significantly increased ultimate stress and Young’s modulus. No significant difference was found in stiffness (Figures 3D–G).

### miR-15b-5p and Fibroblast Growth Factor (FGF) Are the Downstream Targets of circRNA-Ep400

**FIGURE 4:**
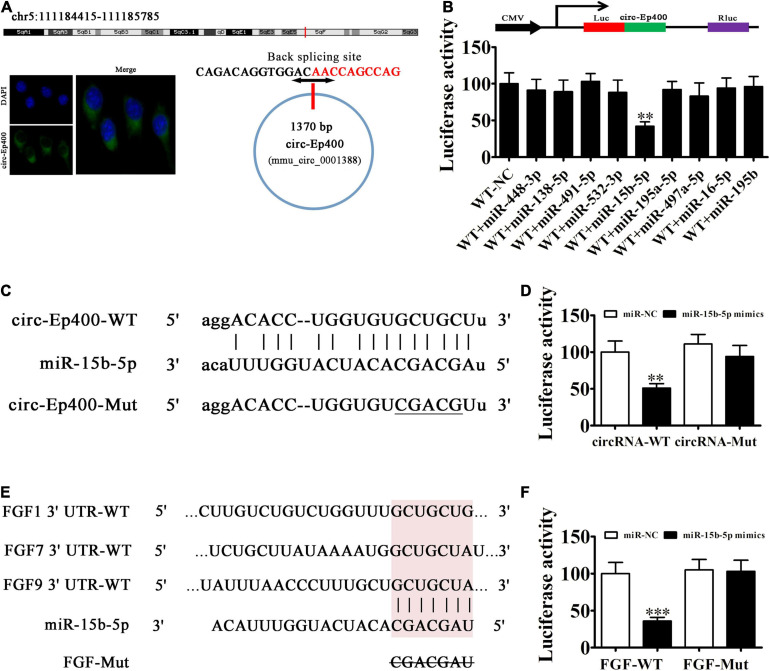
The miR-15b-5p and fibroblast growth factor (FGF) are the downstream targets of circRNA-Ep400. **(A)** Genomic loci and *in situ* hybridization was used to localize subcellular circRNA-Ep400. A red signal indicates back splicing. **(B)** The dual-luciferase reporter assay showing that wild-type and mimic miR-15b-5p co-transfection significantly decreased luciferase activity in HEK293T cells. **(C)** Prediction of miR-15b-5p binding sites in circRNA-Ep400. The mutant version of circRNA-Ep400 is shown. **(D)** Relative luciferase activity determined 2 days after HEK293T cell transfection with the miR-15b-5p mimic/NC or circRNA-Ep400 WT/Mut. Data are expressed as the mean ± SD. ^∗∗∗^*P* < 0.001. **(E)** The miR-15b-5p binding site predictions within the FGF-1/7/9 3′-UTR. The mutant version of 3′-UTR-FGF-1/7/9 is shown. **(F)** Relative luciferase activity determined 2 days after HEK293T cell transfection with miR-15b-5p mimic/NC or 3′-UTR-FGF-1/7/9 WT/Mut. The results are expressed as the mean ± SD. ^∗∗^*P* < 0.01 and ^∗∗∗^*P* < 0.001.

CircRNA-Ep400 originates from circularizing exons from the *Ep400* gene at chr5:111184415-111185785. *Ep400* is comprised of 1,370 bps and the spliced mature circRNA is comprised of 1,370 bps (Figure 4A). FISH assays found that circRNA-Ep400 were predominantly localized to the cytoplasm. An increasing number of studies have confirmed that circRNAs, including miRNA/miR response elements, can connect miRNAs as competitive endogenous RNAs to regulate the expression of target mRNAs (Lin and Chen, 2018; Panda, 2018). Biological information analysis combined with luciferase reporter analysis has shown that circRNA-Ep400 cannot interact with miR-195a-5p, miR-138-5p, miR-448-3p, miR-491-5p, miR-532-3p, miR-497a-5p, miR-16-5p, and miR-195b. However, circRNA-Ep400 only interacted with miR-15b-5p, because miR-15b-5p inhibited luciferase activity in WT cells (Figure 4B). Bioinformatic analyses showed that miR-15b-5p was the downstream target of circRNA-Ep400. To establish a correlation between circRNA-Ep400 and miR-15b-5p, we constructed WT or mutant (MUT) circRNA-Ep400 sequences containing the miR-15b-5p binding sequence inserted into a luciferase reporter vector (Figure 4C). The luciferase reporter vector was then introduced into 293 T cells combined with or without the miR-15b-5p mimic. Luciferase reporter analysis showed that miR-15b-5p inhibited luciferase activity in WT cells, but not in MUT cell lines (Figure 4D), suggesting that miR-15b-5p was the target of circRNA-Ep400.

Bioinformatic analyses verified that FGF1/7/9 was the miR-15b-5p downstream target. To further characterize the correlations between miR-15b-5p and FGF1/7/9, we constructed WT or MUT 3′-UTR-FGF1/7/9 sequences containing the miR-15b-5p binding sequence inserted into the luciferase reporter vector (Figure 4E). The luciferase reporter vector was injected into 293 T cells combined with or without the miR-15b-5p mimic. The luciferase reporter analyses suggested that miR-15b-5p inhibited luciferase activity in WT cells, but not in MUT cell lines (Figure 4F), suggesting that FGF1/7/9 was the target of miR-15b-5p.

### M2 Macrophage-Derived circRNA-Ep400-Containing Exosomes Promote Pro-fibrotic Activities of Tenocytes and Fibroblasts *in vitro*

**FIGURE 5:**
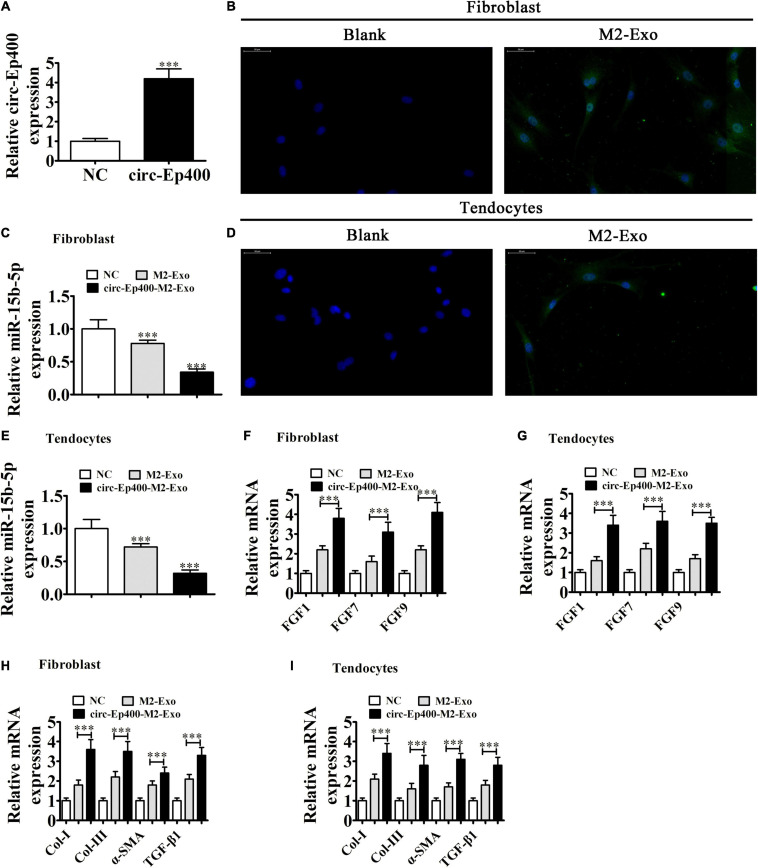
M2 macrophage-derived circRNA-Ep400-containing exosomes promote profibrotic activity in tenocytes and fibroblasts *in vitro*. **(A)** RT-qPCR showing the circRNA-Ep400 expression in exosomes derived from M2 macrophages (NC) or overexpressed circRNA-Ep400 M2 macrophages. The results are expressed as the mean ± SD. ^∗∗∗^*P* < 0.001 vs. NC. **(B)** Fibroblasts were transfected with PKH26-labeled exosomes. **(C)** RT-qPCR showing the miR-15b-5p expression after treatment with exosomes from M2 macrophages or overexpressed circRNA-Ep400 M2 macrophages. The results are expressed as the mean ± SD. ^∗∗∗^*P* < 0.001 vs. the NC. **(D)** Tenocytes transfected with PKH26-labeled exosomes. **(E)** RT-qPCR showing miR-15b-5p expression after treatment with exosomes from M2 macrophages or overexpressed circRNA-Ep400 M2 macrophages. The results are expressed as the mean ± SD. ^∗∗∗^*P* < 0.001 vs. the NC. **(F,G)** RT-qPCR showing FGF1, FGF-7, and FGF-9 expression in both fibroblasts and tenocytes after treatment with exosomes from M2 macrophages or overexpressed circRNA-Ep400 M2 macrophages. The results are expressed as the mean ± SD. ^∗∗∗^*P* < 0.001. **(H,I)** RT-qPCR showing the Col-I, Col-III, and α-SMA, as well as TGF-β1 expression levels in both tenocytes and fibroblasts after treatment with exosomes from M2 macrophages or overexpressed circRNA-Ep400 M2 macrophages. The results are expressed as the mean ± SD. ^∗∗∗^*P* < 0.001.

RT-qPCR detection showed that the expression of circRNA-Ep400 was significantly increased in exosomes derived from circRNA-Ep400 overexpressed M2 macrophages when compared with the NC group (Figure 5A). To determine if macrophage-derived exosomes could be internalized by fibroblasts and tenocytes, we co-cultured fibroblasts and tenocytes with PKH26-labeled exosomes. Immunofluorescence detection showed that PKH26-labeled exosomes were tracked in both fibroblasts (Figure 5B) and tenocytes (Figure 5D). RT-qPCR analysis showed that miR-15b-5p expressions in both fibroblasts (Figure 5C) and tenocytes (Figure 5E) were decreased after treatment with exosomes from M2 macrophages, especially from circRNA-Ep400 overexpressed M2 macrophages. RT-qPCR analysis showed that the expressions of FGF1, FGF-7, and FGF-9 in both fibroblasts and tenocytes were increased after treatment with exosomes from M2 macrophages, especially from circRNA-Ep400 overexpressed M2 macrophages (Figures 5F,G). The RT-qPCR detection also showed that Col-I, Col-III, and α-SMA, as well as TGF-β1, expressions in both tenocytes and fibroblasts were increased after treatment with exosomes from M2 macrophages, especially from overexpressed circRNA-Ep400 M2 macrophages (Figures 5H,I).

**FIGURE 6:**
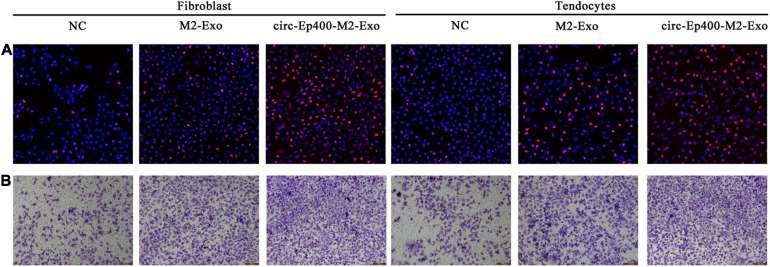
M2 macrophage-derived circRNA-Ep400-containing exosomes promote proliferation and migration of fibroblasts and tenocytes *in vitro*. **(A)** The 5-ethynyl-2’-deoxyuridine assay showing the proliferative ability of both fibroblasts and tenocytes. **(B)** Transwell assays show the migration ability of both fibroblasts and tenocytes.

Edu (Figure 6A) and Transwell (Figure 6B) assays showed that the proliferation and migration ability were increased in both fibroblasts and tenocytes after treatment with exosomes from M2 macrophages, especially from overexpressed circRNA-Ep400 M2 macrophages.

## Discussion

In this study, we found that macrophages infiltrate in peritendinous tissues after TI. Macrophage depletion inhibited peritendinous fibrosis, suggesting that macrophages play an important regulatory role after TI. Immunohistochemical studies showed that the infiltrating macrophages were M1 macrophages. Due to the microenvironment, macrophages are categorized as alternatively activated M2 or typically activated M1 (Ji et al., 2016). M1 macrophages are induced form M0 macrophages by inflammatory stimuli, including lipopolysaccharides, and which are identified by high production of proinflammatory cytokines like IL-1β, IL-12, reactive oxygen species, tumor necrosis factor-α, and iNOS, which suppress tissue recovery after aggravated injury (Benoit et al., 2008; Mege et al., 2011). The M2 phenotype, induced by the Th2 cytokines IL-13 or IL-4, are identified by arginase 1 production and detected in inflammatory zone 1, and which are indispensable for inflammation resolution, tissue repair, and wound healing (Mantovani et al., 2004; Nair et al., 2006; Satoh et al., 2010). An increasing number of studies have found that exosomes secreted from macrophages play an important role in the regulation of the microenvironment (Bardi et al., 2018). Our study also characterized the regulatory role and mechanism of M2 macrophage exosomes after TI.

Our high-throughput sequencing found that circRNAs were abnormally expressed in exosomes from M0 and M2 macrophages. Further study showed that circRNA-Ep400 was highly expressed in M2 macrophage exosomes. CircRNA-Ep400 was localized in the cytoplasm and derived from circularizing exons from the *Ep400* gene, which is located on chr5:111184415-111185785. Previous studies have found that M2 macrophages are a major source of TGF-β1 and platelet-derived growth factor, which, in turn, induce the differentiation of fibroblasts into myofibroblasts (Su et al., 2015; Lipman et al., 2018). In this study we found that M2 macrophage exosome treatment promoted fibrosis by increased Col-I, Col-III, and α-SMA, as well as TGF-β1, expression after TI. Downregulation of circRNA-Ep400 suppressed the therapeutic effect (including tensile strength, ultimate stress, and Young’s modulus of tendons) of M2 macrophage exosomes after TI.

Bioinformatics and luciferase report analyses confirmed that miR-15b-5p and FGF1/7/9 were downstream targets for circRNA-Ep400. High levels of circRNA-Ep400 containing exosome treatment inhibited miR-15b-5p, but promoted FGF1/7/9 expression in both fibroblasts and tenocytes.

An increasing number of studies have reported that FGF1/7/9 plays an important role in Achilles tendon restoration after TI (Reed and Johnson, 2014; Inoue et al., 2015; Guo et al., 2020). High circRNA-Ep400-containing exosome treatment promotes fibrosis by increasing Col-I, Col-III, and α-SMA, as well as TGF-β1, expression in both tenocytes and fibroblasts. High circRNA-Ep400 containing exosome treatment also promoted proliferation and migration in both fibroblasts and tenocytes after TI. Biomechanical tests found that M2 macrophage exosomes, especially high circRNA-Ep400-containing exosome treatment, increased the tensile strength of tendons after impairment.

In conclusion, our study found that exosomes from M2 macrophage promoted peritendinous fibrosis posterior tendon injury via the miR-15b-5p/FGF-1/7/9 pathway by delivery of circRNA-Ep400. The study also confirmed a new mechanism responsible for macrophage-stromal cell communication, revealing a novel role for macrophage-derived exosomes in tendon adhesion, which could lead to new directions in clinical research.

## Data Availability Statement

The original contributions presented in the study are included in the article/Supplementary Material, further inquiries can be directed to the corresponding author/s.

## Ethics Statement

The animal study was reviewed and approved by the Animal Research Committee in Shanghai Jiao Tong University Affiliated Shanghai First People’s Hospital approved the animal protocols.

## Author Contributions

YY and BS conceived the study and wrote the manuscript with feedback from other authors. ZW, MY, ZC, and SL performed the experiments and their analyses. MJ and CY performed the experiments and contributed to the main text. All authors approved the final draft.

## Conflict of Interest

The authors declare that the research was conducted in the absence of any commercial or financial relationships that could be construed as a potential conflict of interest.

## Publisher’s Note

All claims expressed in this article are solely those of the authors and do not necessarily represent those of their affiliated organizations, or those of the publisher, the editors and the reviewers. Any product that may be evaluated in this article, or claim that may be made by its manufacturer, is not guaranteed or endorsed by the publisher.

## Footnotes

1
http://www.clodronateliposomes.org

2
http://www.targetscan.org/

## Abbreviations

circRNA: circular RNA
TI: tendon injury
miRNA: microRNA
3′-UTR: 3′-untranslated region.
