# Supplementary material for: Exosomes From M2 Macrophage Promote Peritendinous Fibrosis Posterior Tendon Injury via the MiR-15b-5p/FGF-1/7/9 Pathway by Delivery of circRNA-Ep400
Source: Front Cell Dev Biol. 2021 Aug 27;9:595911. doi: 10.3389/fcell.2021.595911 (PMC8432299; doi:10.3389/fcell.2021.595911)
Supplement: Supplementary file 2 [file Data_Sheet_1.PDF]

Supplementary materials 2. S1

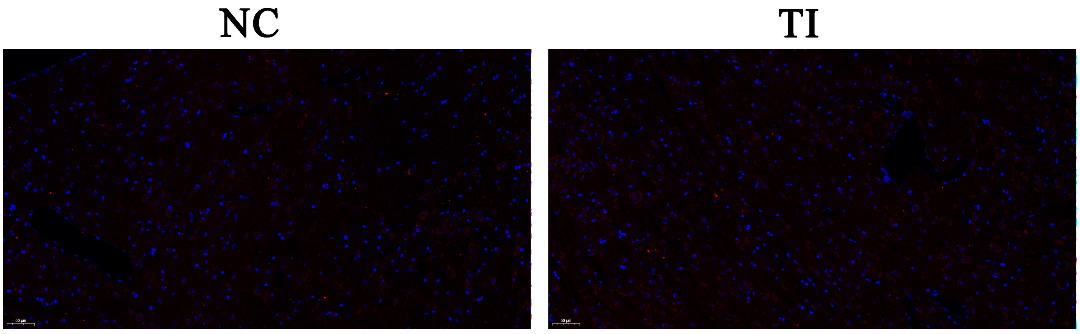

Supplementary materials 2. S2

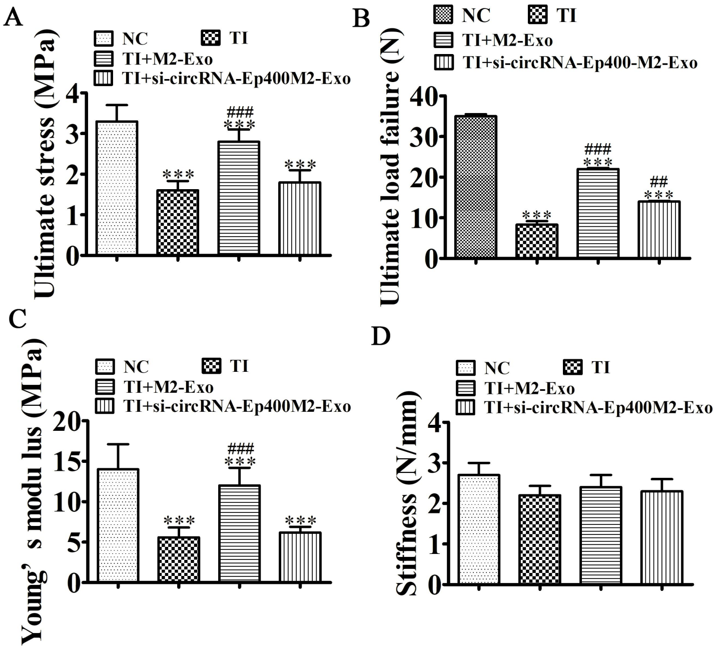

Supplementary materials 2. S3

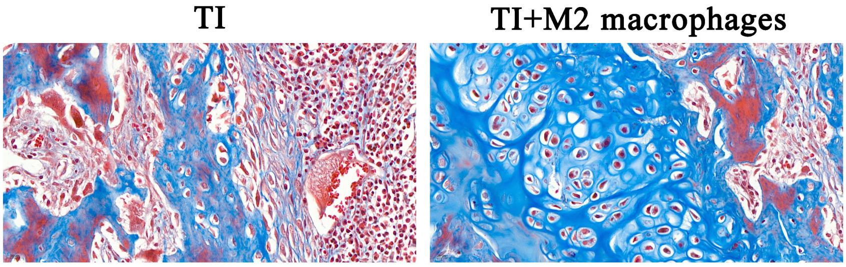

Supplementary materials 2. S4

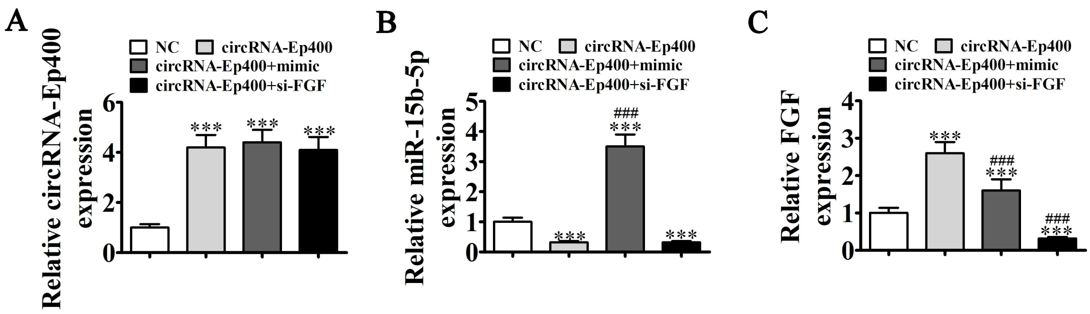
A
NC
circRNA-Ep400
circRNA-Ep400+mimic
circRNA-Ep400+si-FGF
Relative circRNA-Ep400 expression
B
Relative miR-15b-5p expression
C
Relative FGF expression
